# Repply to: Predictive factors for vaginal delivery in pregnant women with previous cesarean section: a case-control study

**DOI:** 10.61622/rbgo/2026rbgo108L23R

**Published:** 2026-10-13

**Authors:** Gabriela Sabbatine Reis, Lucas Casagrande Passoni Lopes, Eleksandra Cibele Gusman Vendramini, Ênio Luis Damaso, Vera Therezinha Medeiros Borges

**Affiliations:** 1 Universidade Estadual Paulista Faculdade de Medicina de Botucatu Botucatu SP Brazil Faculdade de Medicina de Botucatu, Universidade Estadual Paulista, Botucatu, SP, Brazil; 2 Universidade de São Paulo Faculdade de Medicina de Bauru Bauru SP Brazil Faculdade de Medicina de Bauru, Universidade de São Paulo, Bauru, SP, Brazil; 3 Universidade de São Paulo Hospital de Reabilitação de Anomalias Craniofaciais Bauru SP Brazil Hospital de Reabilitação de Anomalias Craniofaciais, Universidade de São Paulo, Bauru, SP, Brazil

Dear Editor,

We sincerely thank the authors for their thoughtful comments and for their generous appraisal of our study. We are pleased that our findings were considered a valuable contribution to individualized counseling for women with a previous cesarean delivery.

We fully agree that improving vaginal birth after cesarean (VBAC) rates requires more than accurate prediction models. Structured prenatal education, maternal empowerment, and institutional preparedness are fundamental to increasing women's confidence in trial of labor after cesarean (TOLAC). In this regard, the "Pregnancy Schools" implemented by the Turkish Ministry of Health represent an interesting strategy that could be adapted to other healthcare systems.

This discussion is particularly relevant in Brazil, which has one of the highest cesarean section rates worldwide. National cesarean rates have remained around 56%, reaching more than 80% in the private sector, far exceeding the levels recommended by the World Health Organization.^(1-3)^ Women with a previous cesarean delivery (Robson Group 5) represent one of the major contributors to the overall cesarean rate in Brazil, reinforcing the importance of promoting safe and evidence-based VBAC whenever appropriate.^(1,2)^

Current FIGO and ACOG recommendations indicate that TOLAC is associated with success rates between 60% and 80% in appropriately selected women, while the risk of uterine rupture after one previous low-transverse cesarean remains low (approximately 0.3–0.7%). Importantly, ACOG emphasizes that prediction models should support, rather than replace, shared decision-making, and that a predicted success probability below 70% should not be considered a contraindication to TOLAC.^(4,5)^ These recommendations are fully aligned with the objective of our study, which was to improve individualized counseling rather than restrict access to TOLAC.

The Brazilian experience also highlights the complexity of balancing maternal autonomy and evidence-based obstetric care. In 2019, the State of São Paulo enacted legislation allowing women to request an elective cesarean section in the public health system. Subsequent analyses demonstrated a temporary increase of approximately 3 percentage points in cesarean rates, mainly due to an increase in primary cesarean deliveries, without significant improvements in neonatal outcomes. Following the revocation of the law, cesarean rates returned to previous levels.^(6)^ These findings reinforce the importance of structured prenatal counseling based on the best available evidence, particularly regarding the real probability of successful VBAC.

Finally, we appreciate the authors’ comments regarding the interdelivery interval. As acknowledged in our manuscript, this variable could not be evaluated because of incomplete documentation in the retrospective medical records. We agree that future prospective studies incorporating this variable, together with structured educational interventions and validated clinical predictors, may further improve VBAC prediction models and facilitate their implementation across different healthcare settings.

Once again, we thank the authors for enriching the discussion on this important topic.
